# The Relationship between Spatial Characteristics of Urban-Rural Settlements and Carbon Emissions in Guangdong Province

**DOI:** 10.3390/ijerph20032659

**Published:** 2023-02-01

**Authors:** Liya Yang, Honghui Zhang, Xinqi Liao, Haiqi Wang, Yong Bian, Geng Liu, Weiling Luo

**Affiliations:** 1 Guangdong Guodi Planning Science Technology Co., Ltd., Guangzhou 510650, China; 2 College of Resources and Environmental Sciences, Hunan Normal University, Changsha 410081, China; 3 School of Geography and Planning, Sun Yat-Sen University, Guangzhou 510275, China

**Keywords:** settlement structures, carbon emissions, spatial planning, GHG emissions

## Abstract

As containers of human activities, both urban and rural built-up settlements play roles in the increment of regional GHG emissions. This study investigates the relationship between the spatial characteristics of different urban-rural settlements and carbon emissions in Guangdong province, China. After estimating the carbon emissions of 21 cities in Guangdong province from 2005 to 2020, this paper constructs a panel regression model based on the STIPRAT model to identify the impact of different types of urban-rural settlements on carbon emissions with controlling socioeconomic factors. The results show that the increase in high-density urban areas and low-density rural built-up areas have a significant positive correlation with carbon emissions. Moreover, the impact of rural built-up settlements is stronger than urban areas. In addition, our results indicate that carbon emission has little correlation with the spatial landscape pattern. This study highlights the importance of rural built-up settlements for understanding regional carbon emissions. Local governments should not only focus on the reduction of carbon emissions in the large urban agglomerations but also need to make a plan for the small and medium-sized towns that are dominated by industries.

## 1. Introduction

The interlocking crises caused by GHG emissions have been the focus of various research fields [1,2,3]. It is well-known that urban areas play a vital role in the increase of energy consumption and GHG emissions [4,5]. Urban areas are aggregations of population, economic activities, goods and services. More than 70 percent of global GHG emissions associated with energy production are related to cities [5,6]. Researchers across disciplines have investigated various factors that could affect GHG emissions in urban areas, such as population [7], income [8,9], transportation [10], and urban forms [11,12]. However, few of these studies explore regional carbon emissions beyond the urban areas. Although some scholars have discussed household carbon emissions and carbon efficiencies in non-urban areas [13,14], a more comprehensive analysis awaits development. Different from western developed countries, in developing countries, especially in Southeast Asia, the population of rural settlements is dense, and mixed agriculture and industrial activities are located in many villages [15,16]. However, the direct and indirect carbon emissions in these rural places are often neglected [16].

Furthermore, the broad classification of urban and non-urban settlements is not enough to present the complex distribution of GHG emissions. From the perspective of urbanization, different types of human settlements are transforming from one to another during the process. The result of the transformation could be various, such as small cities, suburbs, and industrial zones. Which type of built-up settlements generate more GHG emissions still lacks understanding at a regional level. Although scholars have studied carbon emissions at different spatial scales, such as regions [17], provinces [18] and urban areas [19], these administrative spatial scales are insufficient to find out the influential type of settlements. Therefore, a downscaling illustration of different genres of urban-rural settlements is required to comprehensively analyze carbon emissions at a regional level.

Therefore, to fill the above research gap, this paper intends to study the effect of different urban-rural settlements on carbon emissions at a provincial-regional scale, which assumes that both urban and rural built-up settlements play evident roles in the increment of GHG emissions. Hence, we select the Guangdong province of China as the study case, estimate the carbon emissions data from 2005 to 2020 and extract the corresponding spatial characteristics of different built-up settlements, to quantify the relationship between spatial characteristics and GHG emissions. The land proportion of different urban-rural settlements and spatial morphological forms of all built-up settlements are selected to represent the spatial characteristics. Furthermore, we utilize a fixed effects model extended from the STIRPAT model to control the socioeconomic factors and include spatial characteristics as technological variables. The results demonstrate that the area increases of certain types of settlements generate significant influences on carbon emissions. By contrast, the morphological characteristics of urban forms fail to generate an impact on GHG emissions after controlling for sociodemographic factors. Specifically, rural villages in Guangdong are positively associated with GHG emissions due to the allocation of local industries. Although the metropolitan area is the major contributor to emissions in terms of volume, the intensity of carbon emissions is lower than in these rural settlements.

The main contribution of this paper lies in that it analyzes the relationship between different types of urban-rural settlements and carbon emissions at a regional scale from a dynamic perspective. How socio-demography, different built-up settlements, and spatial forms are associated with carbon emissions are comprehensively discussed. It yields an empirical result to highlight the importance of local context for understanding regional carbon emissions, which is vital for the optimization of spatial planning and low-carbon development.

## 2. Literature Review

The fundamental reason for understanding carbon emissions through the spatial perspective is that the physical space is the container of human activities and human activities are the most significant driver of GHG emissions. Among all types of physical space, urban-rural settlements are areas where most GHG emissions originate [4]. Therefore, scholars have tried to analyze the relationship between the characteristics of built-up space and carbon emissions, which includes the type of settlements [20], volume [13], spatial forms [21], and the arrangement of land uses. This section reviews the relationship between built-up settlements and carbon emissions in terms of the above aspects.

### 2.1. Urban-Rural Settlements and Regional Carbon Emissions

The urban settlement accounts for the largest portion of carbon emissions, and hence it is under the spotlight of academic research, just as the statement in the introduction. In terms of the volume of settlements, the expansion of urban settlements brings increases in GHG emissions [9,22]. Moreover, in the same country, GHG emissions also change significantly depending on the city’s size and degree of prosperity [23]. On the other hand, numerous studies focused on the bilateral correlation between urbanization and carbon emissions, which indicated that the positive correlation was not stable and permanent. Martínez-Zarzoso and Maruotti [24] studied the relationship between carbon emissions and urbanization at a global level. They proposed an inverse “U”-shape theory to describe the relationship at different stages of social development. The GHG emissions rose with the urbanization process at the early stage of development. However, the relationship would turn to a negative correlation when urbanization reached a high level. The higher level of urbanization brought a higher utilization efficiency of resources that could reduce carbon emissions to a certain extent [25]. However, the major limitation of this research lies in that it cannot infer the integral regional carbon emissions. As Baiocchi, Creutzig, Minx and Pichler [20] mentioned, “such an approach may suppress both spatial context, non-linear effects, and the interdependence of emission drivers”. There is still a lack of downscaling understanding concerning which type of built-up settlement could generate more GHG emissions.

Regarding other types of settlements, the major focus is the rural household carbon emissions and carbon efficiencies [13,26,27]. In general, household carbon emissions in rural settlements are lower than in urban settlements. However, the GHG emission from private transportation grew faster than urban residents during 1996–2012 in China [27]. Moreover, black carbon emissions (the incomplete combustion of fossil fuels, biofuel, and biomass) also contributed to the GHG emissions significantly in many rural areas of Asia [28]. Zhu et al. [29] studied the carbon emissions of the urban-rural fringe area in East Asia and concluded that the pressure of GHG emissions in these settlements was higher than in the urban center due to the higher production emissions.

So far, few studies have considered rural settlements as a spatial unit to observe GHG emissions. Discussion regarding other types of settlements was also rare. However, the above studies showed the necessity of rural settlements in being considered in research on regional carbon emissions.

### 2.2. Spatial Forms and Carbon Emissions

Regarding carbon emissions and the arrangement of land uses, the most common approach is to utilize land use as the proxy to study the variation of regional carbon emissions [30,31,32]. The spatial utilization of the built-up space–land uses, and spatial forms will affect the efficiency and pattern of regional carbon emissions. Overall, results showed that the spatial distribution of total carbon emissions was mainly from the built-up land and with strong spatial heterogeneity. Zhang and Wu [33] further quantified how the change in built-up land uses impacts carbon emissions through structural equation modeling, indicating that regulating the location, scale, and intensity of land uses can be a feasible approach to promote emission reduction.

However, as Cai et al. [11] stated, most current spatial simulations of carbon emissions based on land uses failed to consider the impact of urban forms. The urban spatial form could reflect the urban traffic network, infrastructure, functional areas, and spatial organization of the population [34]. Kennedy et al. [35] noticed that geophysical conditions and technical factors could influence urban GHG emissions. The design of the urban structure was considered a technical factor in their research. Essentially, the urban form could reflect the degree of diversity and complexity of human settlements. According to the definition of technology, Crawford [36] argued that “technology can also be viewed as a more comprehensive sociotechnical system”, and thus the urban form could be counted as a technical factor in the sociotechnical system.

At present, scholars mainly use the landscape pattern index [21,34,37], network density of roads [38], and population density [39,40] to measure urban forms. Several studies indicated that compact cities could improve carbon emission efficiency [38,39], and a diversified spatial arrangement is a lower-carbon form than a mono-organization [41]. For example, Fang et al. [42] concluded that the increased urban shape complexity tended to generate a positive influence on CO_2_ emissions among 30 provincial capital cities in China. Moreover, the relationship between complex urban spatial forms and carbon emissions is more significant in large cities [12]. However, Wang et al. [43] found that a more polycentric urban form did not have a significant relationship with GHG emissions.

Despite this, few of them explored the relationship at a regional level and combined socioeconomic factors to conduct in-depth research. The true impact of spatial forms on GHG emissions will not appear until other socioeconomic factors are controlled in the model. Therefore, this study is going to consider the spatial form of built-up settlements as a technical factor and enter them into an extended STIRPAT model for discussing the compound effect of spatial form on GHG emissions. Moreover, inspired by the study of Crawford [36], this study considers both genres and forms of human settlements as technical factors in the model because they are the production and representation of historical and current human development.

## 3. Materials and Methods

### 3.1. Study Area and Research Design

Since China’s reform and opening up in the 1980s, urbanization and economic growth have advanced by leaps and bounds, and the total carbon emissions also have increased rapidly. The average annual increasing rate of China’s carbon emissions reached 7.86% from 1997 to 2012 and slowly declined after 2013 [44]. Nevertheless, the total carbon emissions remained at a high level and extended to 10081.34 million tons in 2021, according to the data from International Energy Agency [45]. For enacting effective mitigation strategies, it is necessary to understand the GHG emissions in different regions of China because the territorial and socioeconomic differences are huge within these regions. Therefore, we choose Guangdong province as representative of the most developed region of China to analyze the historical evolution of GHG emissions and its relationship with spatial characteristics of built-up settlements.

**Figure 1:**
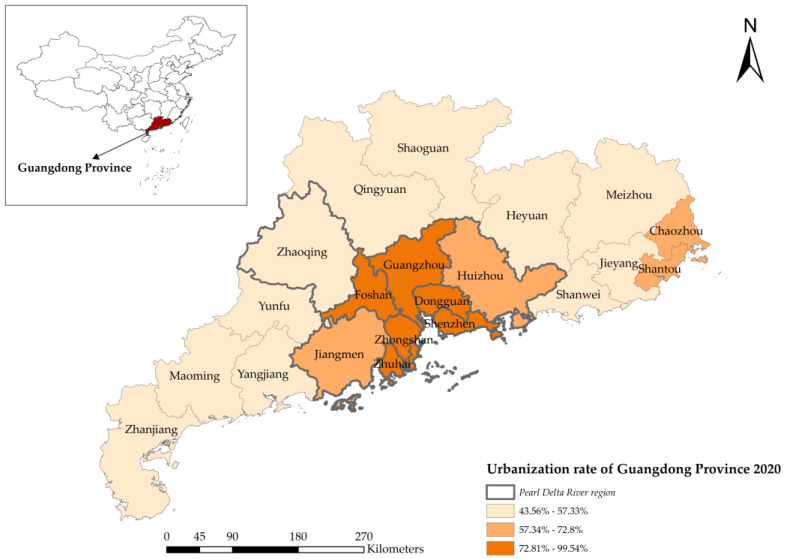
Study area.

As a manufacturing hub of China, the Guangdong province is a coastal province located in the south of China, which consists of 21 cities (Figure 1). The Gross Domestic Product (GDP) retained the first ranking in China for 32 years and reached approximately USD 1.79 trillion in 2021. According to the seventh national census data, the total population was about 127 million, and the average rate of urbanization was 74.63% in 2020. Therefore, Guangdong province is a typical place that can reflect the environmental effect of rapid economic development. On the other hand, the developing differences among cities are notable. The urbanization rate of the Pearl River Delta (Guangzhou, Shenzhen, Foshan, Zhaoqing, Dongguan, Huizhou, Zhuhai, Zhongshan, and Jiangmen) reached 86.28%, and the built-up area exceeded 30% of the administrative area, which is also one of the economic and industrial engines in China. By contrast, the urbanization rate was 60.38% in the eastern region of Guangdong, 45.18% in the west, and 50.80% in the northern part. Such a regional difference is helpful in understanding how spatial characteristics are correlated with GHG emissions.

**Figure 2:**
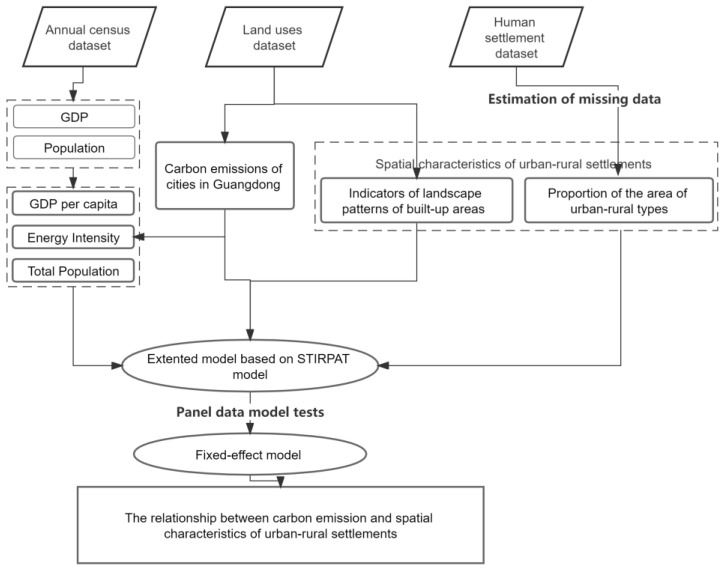
Research framework.

This study quantifies the relationship between carbon emission and spatial characteristics of urban-rural settlements at a city level based on the administrative boundary of Guangdong province. A 15-year land uses dataset is used to estimate the carbon emissions and calculate the morphological characteristics of all built-up areas. A global human settlement dataset is utilized to extract the variations of different types of urban-rural settlements in Guangdong province. The research design (Figure 2) follows three main steps:

1. utilizing land uses data to access carbon emissions (Section 3.3);
2. extracting spatial characteristics in terms of different urban-rural types and morphological forms of all built-up settlements (Section 3.4);
3. quantifying the relationship by a fixed-effect model (Section 3.5).

### 3.2. Data Collection and Processing

The land uses data that we use is from the Institute of Geographic Sciences and Natural Resources Research(data source: CNLUCC [46]). It contains 26 categories of land uses with an accuracy of 1 × 1 km, such as paddy fields, shrubbery, rural settlements, and urban land. We obtained six years of data: 2005, 2010, 2013, 2015, 2018, and 2020. The socioeconomic data is extracted from the Guangdong Statistical Yearbook, which is released every year by the Statistics Bureau of Guangdong province [47]. The granularity of statistics consists of a city level, a regional level and a provincial level. In this study, we used city-level data in the corresponding years of land use datasets. The total population is defined by the number of permanent residents (that is, people who have lived in the place for more than six months).

The human settlement dataset is extracted from the GHSL (Global Human Settlement Layer) of the European Commission [48], from which categories and standards of classifications are listed in Table 1. Since the two datasets have the same spatial accuracy, the spatial unit of the calculation is set as a 1 km^2^ grid in all cities of Guangdong. However, as the GHSL data is only released every five years, we estimate the data of the missing years (2013, 2018) by the interpolation method:

(1) $$TA_{i}=TAB_{i}\times {\left(1+r_{i}\right)}^{n}\;$$

where $TA_{i}$ is the area of type *i* settlement within the administrative area of a city; $r_{i}$ is the average changing rate of one type of human settlement in five years, and *n* is the number of years. $TAB_{i}$ is the area of type *i* in the base year. For example, the area of UC in 2013 is estimated by the average variation of UC from 2010 to 2015, in which 2010 is the base year.

### 3.3. Estimation of Carbon Emissions

Following the energy carbon emission coefficient method of the IPCC inventory [49], this paper estimates the carbon emissions of each city in Guangdong province by summing the energy consumption on five types of land use: industry, transportation and storage, cultivated land, urban built-up area, and rural built-up area. Utilizing the energy balance sheet of Guangdong Province in the corresponding year, we estimate carbon emissions of energy consumptions on different land uses. The formula is expressed as:

(2) $$GHG=\sum_{it}E_{it}\times \beta_{it}$$

where *GHG* is the total amount of carbon emissions; *t* is the year, *i* is the energy type; *E_i_* stands for the energy consumption of *i* type of energy (converted to standard coal), and *β* is the carbon emission coefficient, which is listed in Appendix A Table A1. The final carbon emissions of a city are the sum of GHG of the above land uses within its administrative boundary.

### 3.4. Indicators of Spatial Characteristics of Urban-Rural Settlements

The measure of spatial characteristics of urban-rural settlement in this study is divided into two aspects: the urban-rural type and the morphological form of all built-up settlements. Types of human settlement space refer to the division of human settlement according to the population density and the built-up settlement type, such as urban areas with a high density of population, suburban areas, and rural areas. In order to avoid the effect of proportionality between socioeconomic factors and built-up factors, this paper chooses the form of percentage as the characteristics of urban-rural settlement in a city. The calculation is expressed as:

(3) $$PT_{i}=\sum TA_{i}/Total\;Area$$

in which PT represents the proportion of an urban-rural type, and *Total Area* is the area of a city’s administrative divisions in 2022.

The morphological characteristics of human settlement refer to the spatial forms of all urban-rural built-up areas at a city level, which is analyzed by the landscape pattern index [50] using the land uses dataset. The landscape pattern index is a set of quantitative indicators that reflect the composition of landscape patterns. Following previous studies [21,38], this paper utilizes six landscape indicators to describe the spatial form pattern (Table 2). The calculation process is completed by FragStats software (version 4.2).

### 3.5. Fixed Effects Model Based on STIRPAT Model

York et al. [51] proposed the STIRPAT model (The Stochastic Impacts by Regression on Population, Affluence, and Technology) on the basis of the IPAT model. The STIRPAT considers the impact of population, wealth, and technological factors on the environment and solves the problem of proportionality between variables [52]. This model is one of the most commonly used methods for studying the relationship between carbon emissions and socioeconomics [53,54]. Moreover, scholars often extended the basic model by adding more contextual variables or control variables for investigating more relationships between carbon emissions and other factors, such as human activities [55] and urbanization [8]. The basic form of the model is expressed as:

(4) $$I=aP_{j}^{b}A_{j}^{c}T_{j}^{d}e_{j}\;$$

Among them, *I* is the environmental impact pressure, *P* is the population, *A* is the wealth, *T* is the technical influence factor, *a* is the model tensor parameter, *b*, *c*, and *d* are the impact parameters to be estimated, *e* is the error term, *j* is the corresponding spatial observation unit. In the field of carbon emissions, the environmental impact usually corresponds to the number of carbon emissions; *P* is the total population, and *A* is the GDP per capita. The logarithmic form is usually used for transforming the model into an addictive model:

(5) $$LnI=a_{0}+bLn\left(P_{j}\right)+cLn(A_{j})+d\left(LnT_{j}\right)\;$$

Since it is difficult to define the proxy of the technology influencing factor *T*, scholars have many different interpretations. For example, Cao et al. [56] considered *T* as energy intensity, and Ji and Chen [57] used the share of industry output to approximate the *T* factor. Liu, Zhou and Wu [7] combined the share of added values of industry and the industry energy intensity (expressed as industry energy use per unit GDP) to construct the *T* factor. As mentioned in Section 2.2, Crawford [36] argued that the urban spatial form should be considered as the *T* factor.

Since the influence of technology is so complex, this study decided to utilize energy intensity and spatial characteristics as technical factors in the model. The former is more related with the economic and industrial progress, the latter is more correlated with the evolution of built-up space. The EI is the GHG emissions per GDP, which is defined as:

(6) $$EI=\frac{GHG}{GDP}\;$$

Meanwhile, we take the population, per capita GDP, and EI as control variables to analyze the effect of spatial characteristics of built-up settlements on GHG emissions. Indicators of spatial characteristics enter into the extended model as technological parameters. The model is adapted as follows:

(7) $$LnC=a_{0}+bLn\left(P_{j}\right)+cLn(A_{j})+d\left(LnEI_{j}\right)+\beta \left(PT_{j}\right)+\mu \left(LS_{j}\right)+\varepsilon_{j}\;$$

where *j* is the city, *C* refers to the GHG emissions; P is the total population of the city, and A is the GDP per capita. *PT* represents variables of the percentage of different human settlements, *LS* is the indicator of spatial landscape form, and *ε* is the error term. Table 3 describes the time interval and basic statistics of all variables in the model.

**Figure A1:**
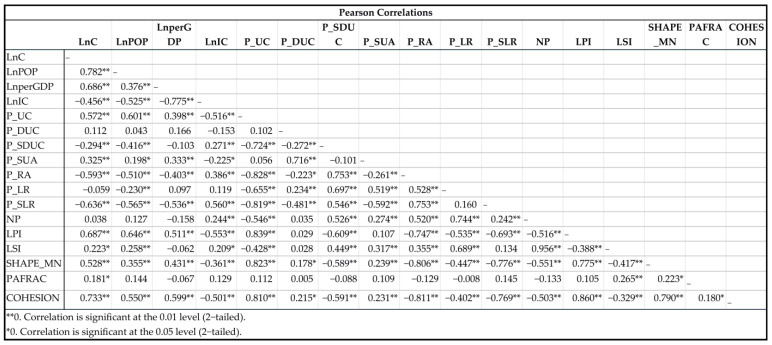
The Pearson correlation between variables.

The next step is to determine the form and effect of the panel data model. Firstly, it is not necessary to examine the cointegration of the panel data in our model since the number of observations is larger than the number of the period. The regression will give a consistent estimation in this case [58]. Secondly, we use LSDV (least-squares dummy variable) regression to perform an F-test on individual dummy variables for determining the form of model-mixed cross-sectional regression (Pooled OLS) or variable coefficient model. The *p*-value of the F-test is less than 0.05, and thus the variable coefficient model should be conducted. The Hausman test is used to test the effects of the mode-random or fixed, which it turned out has rejected the null hypothesis. Therefore, according to the result of these statistical tests (Table 4), the entity fixed-effects model is adopted. In addition, the model uses the cluster–robust estimator to reduce the heterogeneity. The formula then becomes:

(8) $$LnC_{jt}=\alpha_{j}+\beta_{1}Ln\left(P_{j}\right)+\beta_{2}Ln(A_{j,t})+\beta_{3}Ln(EI_{j,t})+\gamma_{i}PT_{j,t}^{i}+\mu_{i}LS_{j,t}^{i}+\varepsilon_{j,t}\;$$

where *β, γ*, and *μ* are terms are coefficients for independent variables, *t* represents time;$\;\alpha_{j}\;$ is the unknown intercept for each entity (that is, cities in this study); $PT^{i}$ refers to the *i th* PT variables, and $LS^{i}$ is the *i th* indicator of landscape patterns. To reduce the collinearity and heterogeneity (see the Pearson correlation test in Appendix A Figure A1, variables of spatial characteristics are introduced into the model one by one. All tests and calculations regarding the model are executed by STATA software (version 16).

## 4. Results

### 4.1. Evolution of Carbon Emissions in Guangdong Province

**Figure 3:**
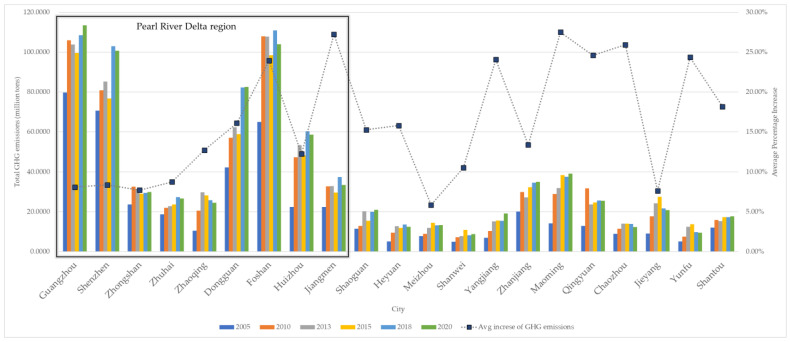
Temporal trends of total carbon emissions in cities of Guangdong.

Figure 3 displays the carbon emissions of cities in Guangdong Province in the last 20 years. The region with high carbon emissions was mainly concentrated in the Pearl River Delta region. Guangzhou, Foshan, Shenzhen, and Dongguan were the main contributors to carbon emissions in Guangdong. The total GHG emissions in the rest cities of Guangdong were relatively low, which just accounted for 30% of the province. However, cities with higher average increasing rates of carbon emissions (over 20%) were located out of the Pearl River Delta region, such as Maoming, Qingyuan, Zhaoqing, and Yangjiang.

**Figure 4:**
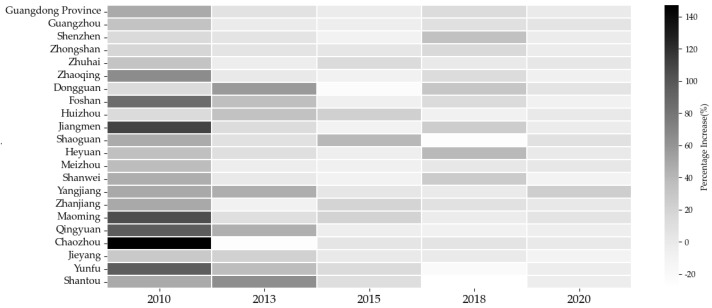
Percentage increase of GHG emissions. Note: the percentage increase is based on the last recorded year. For example, the increment rate of 2010 is based on 2005, et cetera.

In general, 2005–2010 was the period of the fastest growth in carbon emissions. Compared with 2005, GHG emissions in Guangdong province increased by 147% in 2010 (Figure 4). After that, the increasing rate tended to fall to less than 20%, compared to the previous period, even appeared a negative increase in Yunfu, Shantou, and Shaoguan in 2018. Although the increasing rate of Guangzhou, Shenzhen, Foshan, and Dongguan was lower than other cities, their sizeable volume of carbon emissions that led to the increment was still huge.

### 4.2. Transition of Urban-Rural Types of Settlements in Guangdong Province

**Figure 5:**
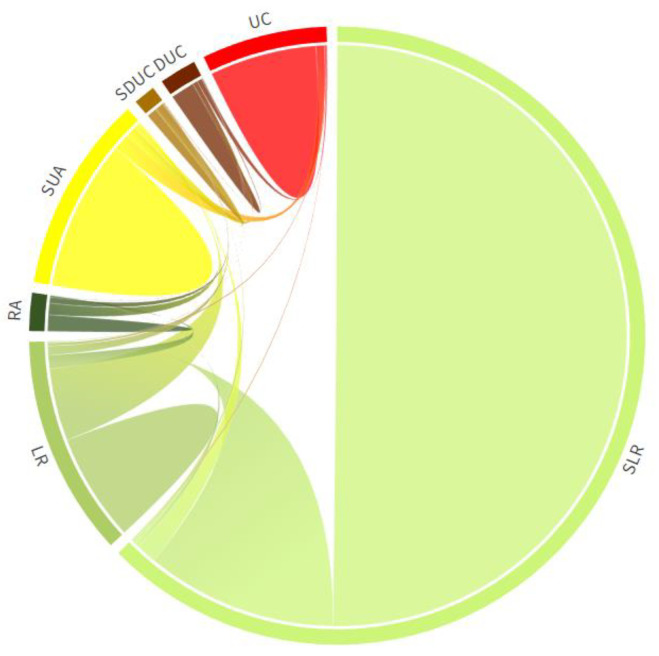
Chord diagram of urban-rural types in Guangdong Province from 2005 to 2020.

Leveraging the variation of areas of different urban-rural settlements in the study period, we can figure out the direction and size of urbanization in Guangdong. This section only discusses the difference between the start year (2005) and the final year (2020) because of the space limitation. The chord diagram (Figure 5) shows the direction of transformation between different urban-rural settlements in Guangdong Province from 2005 to 2020. The general trend was those rural built-up settlements transformed into urban areas. It coincided with the process of urbanization and the agglomeration of the population in Guangdong province. The average urbanization rate of Guangdong increased from 60.68% in 2005 to 74.15% in 2020. In the same period, about 50% of LR (low dense rural region) and RA (dense rural region) converted into small and medium towns (SDUC) or were absorbed by large dense urban areas (UC) as suburban areas (SUA). In rural types, 21.2% of the very low-density rural region (SLR) has transformed into the low dense rural region (LR). Meanwhile, a portion of LR also has become RA during the past 15 years. Amid urban types, the suburbs and semi-urbanized regions (SUA) have been absorbed by medium-to-high-density urban areas (UC and DUC). Compared to 2005, all surfaces of urban types have expanded, especially the SUA, whose area increased by nearly 49%. The specific number could be found in the transformation matrix (see Appendix A Table A2).

**Figure 6:**
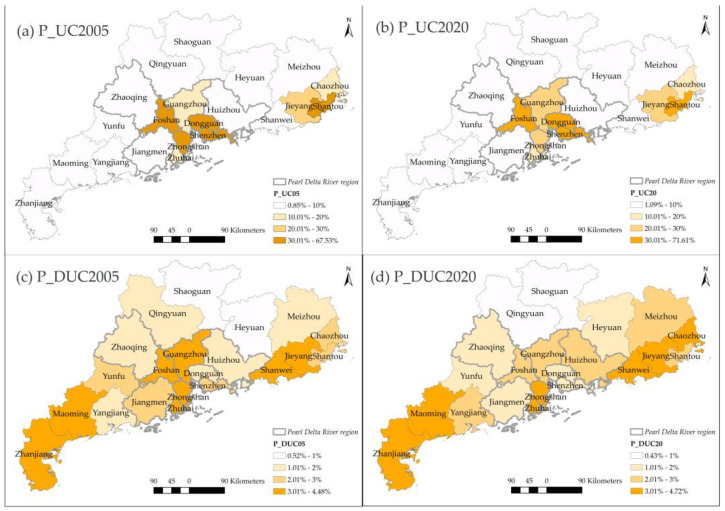
The spatial distribution of P_UC and P_DUC in 2005, 2020.

Secondly, in terms of differences in urban-rural types among cities, an evident heterogeneity can be observed in Guangdong. Figure 6a,b indicate that UC is mainly distributed in the Pearl Delta River region and Shantou. The latter belonged to one of the special economic zones of China established in 1981. Within the Pearl Delta River region, compared with 2005, UC became larger in Guangzhou and shrunk in Zhongshan in 2020. One possible explanation was the competition of cities in the Pearl Delta River region led to Zhongshan’s “degradation”. The GDP of Zhongshan has fallen from fifth to ninth in Guangdong province. The DUC only increased by 5% from 2005 to 2020 in terms of the total amount. The P_DUC (Figure 6c,d) mainly increased in cities located in the eastern region of Guangdong, such as Meizhou, Chaozhou, and Heyuan, while it decreased in the Pearl Delta River region.

**Figure 7:**
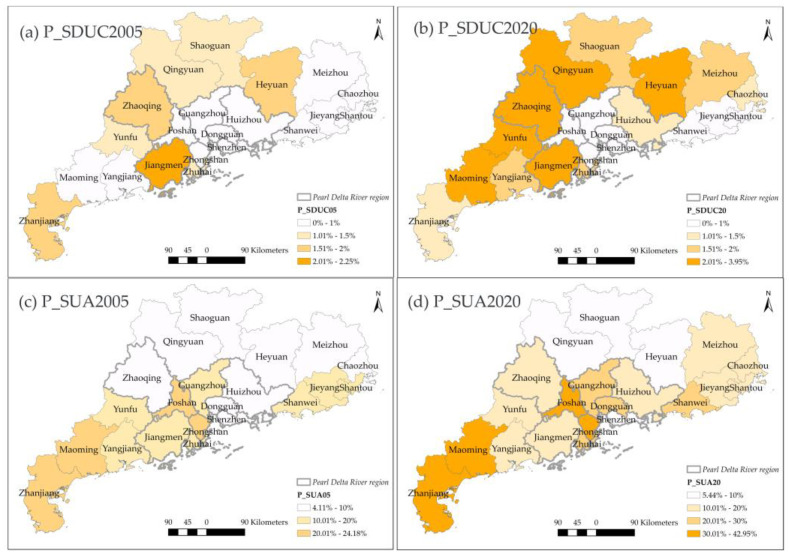
The spatial distribution of P_SDUC and P_SUA in 2005 and 2020.

The SDUC can be interpreted as towns as small cities according to its technical definition (Table 1). The P_SDUC (Figure 7a,b) mainly increased and distributed in the northwestern side of Guangdong, including Maoming, Yunfu, Zhaoqing, Zhanjiang, Qingyuan, and Jiangmen. Although the total surface of SUA dramatically increased, the percentage of SUA did not increase in all cities (Figure 7c,d). The P_SUA did not change significantly in Qingyuan, Shaoguan, and Heyuan, which took a lower P_SUA in 2005. By contrast, cities with more SUA areas in 2005 further expanded their proportion of SUA in 2020, such as Maoming, Zhanjiang, Foshan, and Zhongshan.

**Figure 8:**
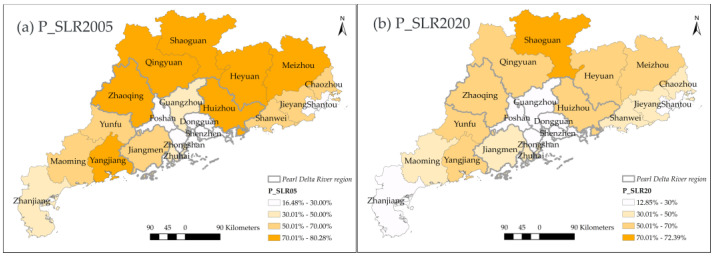
The spatial distribution of P_SLR in 2005,2020.

Regarding the rural space, the disappearance of very low-dense rural areas (SLR) occurred in most of the cities of Guangdong (Figure 8), though the northern part of Guangdong still retained a higher P_SLR in 2020. Dongguan had the smallest P_SLR in 2005 and further decreased by about 3.6% in 2020. Conversely, the rest cities with higher P_SLR dropped by 12% on average. The largest descent of P_SLR happened in Huizhou, where SLR mainly transformed into a suburban or peri-urbanized area (SUA). Similarly, other cities also did not transform into metropolitan areas except Guangzhou. As the provincial capital, master planning has designed Guangzhou to develop into an international metropolis. Therefore, the destination of its major land conversion was UC.

**Figure 9:**
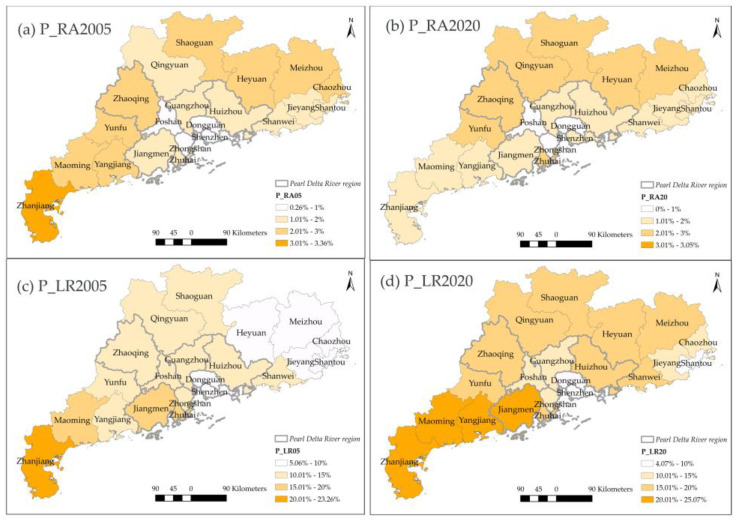
The spatial distribution of P_RA and P_LR in 2005,2020.

The rural settlement also tended to aggregate to the denser rural settlements (RA and LR). However, just as Figure 5 performed, nearly half of RA was transformed into urban types, and thus the P_RA maintained the same level in 2020 (Figure 9a,b). Conversely, the P_LR significantly increased in 19 cities except for Shenzhen and Zhuhai (Figure 9c,d). Heyuan, Meizhou, and Yangjiang increased by over 8%, and the following were Jiangmen and Zhaoqing. These cities also held a higher rise in GHG emissions. To sum up, it could conclude that the metropolitan area concentrated in the Pearl Delta River region, especially in Guangdong, Foshan, Shenzhen, and Dongguan.

To sum up, the urbanization of Guangdong province varied in different cities. The transformation of settlement type was not only the large urban area. In fact, the expansion of SUA and SDUC was evident in many cities. Moreover, the increase of rural settlements also significantly increased. It indicated that the urban settlements might not be the single genre that was related to GHG emissions. Therefore, the next section explored the relationship in detail.

### 4.3. Carbon Emission and Spatial Characteristics of Urban-Rural Settlements

Table 5 and Table 6 list all stable models and results with the same control variables (the natural log of population, GDP per capita, and energy intensity). Model_1 in Table 5, as the primary form of STIRPAT, is the base model for comparison. As a result, the spatial characteristics seem to be considered separately due to the high collinearity between landscape patterns and urban-rural types, though we have tried all possible combinations of morphological and genre variables. The compound effect is discussed in Section 4.3.2. In general, the impact of landscape patterns on GHG emissions was insignificant or very low. Conversely, the type of built-up space has a stronger impact on carbon emissions.

#### 4.3.1. Urban-Rural Types and GHG Emissions

The high R^2^ value of the base model indicates that sociodemographic factors are the main drivers of carbon emissions. Moreover, the results from Model_2 to Model_6 confirm that indicators of built-up settlements perform a significant and positive impact on GHG emissions because the R^2^ of these models is higher than the base model. Specifically, the percentage of large and high-density urban areas (P_UC), low-density rural built-up settlements (P_LR), and urban, suburban areas(P_SUA) have a stable and significant positive effect in all models, which means that the increment of these types is positively associated with the GHG emissions in Guangdong regardless of socioeconomic factors. In other words, the increases in carbon emissions are more correlated with these three types of built-up settlements in Guangdong. The P_DUC, P_SDUC, and P_RA only account for a low proportion in all cities (see Section 3.2), and thus their effects are not significant in models (see Model_4 and Model_5) at the 0.05 level. The P_SLR is excluded from models due to its highly negative correlation with GHG emissions.

Model_3 obtained the highest R^2^ value (0.811) among models, indicating that it is the most explanatory model regarding GHG emissions. The R^2^ of Model_6 is slightly higher than Model_3. However, P_RA loses significance in Model_6. If we remove P_RA, Model_6 will be the same as Model_3. Model_4 is a similar circumstance. Regarding Model_2 and 3, Model_2 would be the best fitting model if we follow the strictest criteria, that is, significance at 0.01 level. In that case, P_SUA would become an insignificant factor. However, as stated in Section 3.2, the area of SUA in 2020 expanded by 49% compared to 2005. From this point of view, the model that includes P_SUA can better explain the regional carbon emissions. Therefore, we select Model_3 as the final model for further analysis.

The coefficient of P_LR is the highest among other urban-rural types in Model_3, which means that a higher percentage of low-dense rural settlements is associated with higher carbon emissions. The rest models also appear to have a similar relationship. Such a finding could be interpreted from two aspects. Firstly, as previous studies pointed out [21,39], the carbon emission efficiency of compact high-density urban areas (UC) was significantly higher than that of low-density rural built-up areas. Secondly, another possible explanation is that many village-level industrial parks have emerged in Guangdong Province since the 1980s [59]. They are mainly distributed in the Pearl River Delta, western Guangdong, and northern Guangdong. It was coincident with the increases of LR in 19 cities of Guangdong during this period. The seven cities with over 20% of the average increasing rate of GHG emissions also had a higher percentage of LR in 2020, except Foshan and Chaozhou. The P_LR of Foshan was not high because of its high percentage of urbanization. Moreover, Foshan has started to upgrade these village-level industrial parks since 2000 [60]. Another supportive evidence is the increment in the population. According to the 2021 census of Guangdong province, the increase of population in the rest of Guangdong was slower than in the Pearl Delta River region. Some cities even appeared the decrement of population, such as Heyuan, Shanwei, and Zhanjiang.

P_UC is the second influential type in the model. The correlation between large dense urban areas and carbon emissions was confirmed by many studies [5,8,11]. Except for the possible reason above, another possible explanation that the influence of P_UC is less than P_LR is the heterogeneity of spatial distribution of urban-rural types. Shenzhen, Guangzhou, and Shantou are cities with the largest increase in P_UC. However, the total carbon emission of Shantou was not as high as the two cities. The urban size of Shantou is only half of Shenzhen, though its increased percentage of PC is high. Regarding the P_SUA, it is the most increased category in all urban-rural types, while its relationship to GHG emissions is not the most prominent (Model_3 and 6). One possible reason is the function of SUA in these cities. The suburban or peri-urbanized areas are usually constructed with more dwellings and fewer manufacturers because the housing market is profitable in China. Furthermore, the density of the population is lower than in the UC, and thus the household emissions are less than in the urban center.

According to the models’ results, at the provincial level, the route and size of urbanization and economic development are different in cities, which lead to the contribution of different settlement type in GHG emissions are different. The impact of urban settlements actually was less than the rural settlements in Guangdong due to its local conditions. Such a complex local context consists of land policies, economic triggers, competitions among local governments, and so on. However, the detailed reason for the development mode in Guangdong province is beyond the aim of the paper.

#### 4.3.2. Spatial Morphological Forms and GHG Emissions

Table 6 shows that the impact of landscape patterns on GHG emissions was not statistically significant after controlling socioeconomic factors, though both Model_7 and Model_8 exhibit a slightly higher R^2^ value than the base model. The number of built-up patches (NP) was not significant in Model_7 at the 0.05 level. The complexity index (LSI) of built-up patches of human settlements only has a slight impact on the carbon emission in Model_8.

Although Yuan, Guo, Leng and Song [21] and Ou, Liu, Li and Chen [38] concluded that the landscape pattern could affect carbon emissions in the metropolis and middle-small cities, they only discussed the landscape patterns in urban and failed to add the socioeconomic factors into the model. Model_9 tries to explore the compound effect of both spatial indicators by adding two stable variables of urban-rural types in previous models. However, LSI becomes insignificant because P_UC and P_LR are more influential factors, which could further support the conclusion that the GHG emission is more correlated with genres of built-up types than the landscape forms.

## 5. Discussions and Policy Implications

Empirically, we analyze the spatial characteristics of 21 cities in Guangdong province and investigate their correlation with carbon emissions from 2005 to 2020. Nine fixed-effect models are estimated for validating the relationship under different combinations of variables. The results show that different types of built-up settlements are significantly correlated with GHG emissions. Conversely, the spatial form merely has a limited influence on carbon emissions, which is contrary to some previous studies, as we stated in Section 4.3.2.

As the carrier of human activities [61,62], the GHG emissions are not evenly distributed in the built-up space due to the density of the population, the function of the settlement, and other external influences. In the context of Guangdong province, the continuous expansion of built-up areas, mainly at the expense of the very low density of the rural region (SLR), corresponds to the increase of GHG emissions in general. The simplified relationship between urban settlements and GHG emissions has been confirmed by previous studies [37,57].

Regarding the increases in GHG emissions, the Pearl Delta River region contributes to 70% of the total emissions in Guangdong. It is also the region with the highest degree of urbanization. From 2005 to 2020, the population increased from 454.7 million to 782.3 million in the region, according to the census. However, cities with a high increase rate are located out of the region, such as Maoming, Qingyuan and Yangjiang. These cities share two common points: (1) they are still in the process of urbanization; (2) many factories are built or transferred from the Pearl Delta River region due to government policy. Therefore, it could conclude that the emission increase is mainly caused by the socioeconomic development in the region. A downscaling analysis based on different settlements is beneficial for understanding the dynamic spatial distribution of GHG emissions.

Furthermore, the effects of the expansion of these settlements are different. P_LR is the most influential type under different model assumptions. As stated in Section 4.3.1, it is related to the industrial development in villages and towns of Guangdong and the regional contexts. Although many studies have investigated GHG emissions in China at different levels and from different perspectives [9,22,42,44], they did not notice the role of rural settlements in carbon emissions during the development of industrialization and urbanization. Meanwhile, such a finding indicates that the carbon emission efficiency of high-density urban areas is significantly higher than that of rural built-up areas, which is also observed by Fan, Liao, Liang, Tatano, Liu and Wei [14] at the national level and Zhu, Zhang, Gao and Mei [29] in many cities of East Asia. Other built-up spaces, such as RA, SDUC, and DUC, do not perform statistical significance in the model because their variation and proportion of the total area are smaller than other types.

Therefore, according to the result, for the governance strategy of emission reduction, Guangdong province should not only focus on the reduction of carbon emissions in the large urban agglomerations but also need to make a plan for the small and medium-sized towns that are dominated by industries. As a policy tool, spatial planning can intervene in the reduction of GHG emissions by arranging land resources, setting protective regulations, and other administrative and market-based strategies [63,64,65]. Different emission reduction strategies should be formulated according to different built-up types and local scenarios. For high-density urban areas, the area of the built-up region should be controlled rigidly, while the improvement of carbon emission efficiency should be the focus in rural built-up areas. Moreover, it is necessary to strengthen the regulation and surveillance of GHG emissions beyond the urban areas, especially in the Pearl River Delta and the western and northern regions of Guangdong. Spatial-explicit assessments of GHG emissions should be adopted in these areas for selecting priority areas for emission reductions and developing a suitable plan to upgrade the industry.

## 6. Conclusions

Leveraging the extended STIPRAT model, this study takes socioeconomic conditions and carbon emission intensity as control variables and constructs a fixed-effect regression model to discuss the role of different urban-rural settlements and spatial forms in GHG emissions. Our result confirms that the increase of high-density urban areas (UC), suburbs (SUA), and low-density rural built-up areas (LR) has a significant positive correlation with carbon emissions. On the contrary, the built-up landscape patterns fail to generate a significant effect on the GHG emissions in Guangdong province.

The effects of different settlements on carbon emissions in Guangdong also showed an apparent heterogeneity across cities. Even though Guangdong is one of the most developed regions in China, the unbalanced development caused the different forms of settlements among cities. The emission effect of urban settlement growth mainly happened in the Pearl Delta River region. The effect of LR expansion could be observed in cities where many industries were located. These results demonstrated that improving the strategy of land use and upgrading village industries might still be necessary and urgent to mitigate GHG emissions.

Overall, we have shown how and why GHG emissions differ for cities in Guangdong province in the last 15 years. The downscaling methodology described the important types of urban-rural settlements regarding GHG emissions. The result may allow policymakers to understand and potentially reduce emissions in Guangdong. However, this study is limited by the uncompleted dataset. We are unable to deepen the analysis due to the lack of data in villages. In addition, the current datasets of estimation of GHG emissions are hard to compare to each other, which indicates that the result cannot be validated by other datasets at a small scale, though the general trending is the same. Nevertheless, as urbanization is still ongoing and the environmental pressure is still tremendous in China, the comprehensive analysis regarding land policy, the transformation of rural settlements, and GHG emissions should be further analyzed in future work.

## Figures and Tables

**Table 1:** Classification of urban-rural types.

Category of Urban-Rural Settlement	Classification Standard (Unit: 1 km^2^ Grid)			
	Population Density Constraint “>“ (Person/km^2^)	Block Total Population Constraint “>“ (Total Person)	Built-Up Area Constraint “>“ (km^2^)	Spatial Constraints
Large dense urban area (UC)	1500	50,000	0.5	4-connectivity cluster
Medium Dense Urban Area (DUC)	1500	5000	0.5	4-connectivity cluster
Low to Medium Dense Urban Area (SDUC)	300	5000	0	(1) 8-connectivity cluster; (2) Distance to UC or DUC > 3 km
Suburban or peri-urbanized area (SUA)	300	5000	0	(1) 8-connectivity cluster; (2) Distance to UC or DUC < 3 km
Dense Rural Area (RA)	300	500	0	8-connectivity cluster
Low Dense Rural Areas (LR)	50	0	0	None
Very Low Dense Rural Regions (SLR)	0	0	0	land area > 50%

Source: adapted from Schiavina, Melchiorri, Pesaresi, Politis, Freire, Maffenini, Florio, Ehrlich, Goch and Tommasi [48].

**Table 2:** Landscape pattern indicators and interpretations.

Landscape Pattern Index	Meaning	Value Range
Number of built-up spatial patches (NP)	Describe the degree of fragmentation of built-up patches. The larger number of NP is, the higher the degree of fragmentation of built-up spatial forms.	Integral number
Largest Patch Index (LPI)	The proportion of the largest patch of a continuous built-up patch in the entire built-up area.	[0, 1]
Landscape Shape Index (LSI)	Measure the irregularity index of built-up space. The larger LSI indicates the more complex form of built-up area.	≥1
COHESION	Measure the aggregation degree of built-up patches. COHESION increases as the patch aggregates in its distribution.	(0, 100]
Perimeter Area Fractal Dimension (PAFRAC)	Measure the complexity of spatial form. The larger the value, the more complex the spatial form.	[1, 2]
Mean Shape Index (SHAPE_MN)	Measure the complexity of spatial form. The larger the value, the more complex the shape of this type of patch.	>0

**Table 3:** Descriptive statistics of variables.

Variables	Mean	Std. Dev.	Min	Max	Type	Data Source
A	Mean	Std. Dev.	Min	Max	Dependent variables	CNLUCC
LnC	7.805	0.795	6.202	9.337		Census
LnPOP	6.108	0.556	4.953	7.536	Control variables	
LnPerGDP	1.158	0.898	−0.956	2.753		
LnEI	0.348	0.462	−1.011	1.377	PT variables	GHSL
PSD_SLR	0.482	0.204	0.129	0.803		
PSD_LR	0.135	0.047	0.037	0.251		
PSD_RA	0.017	0.008	0.000	0.034		
PSD_SUA	0.149	0.079	0.041	0.430		
PSD_SDUC	0.011	0.008	0.000	0.039		
PSD_DUC	0.025	0.011	0.004	0.049		
PSD_UC	0.169	0.203	0.008	0.716	LS variables	CNLUCC
NP	149.079	105.307	20.000	488.000		
LPI	29.440	24.256	4.745	94.474		
LSI	13.668	3.886	7.661	24.262		
SHAPE_MN	1.186	0.141	1.056	1.726		
PAFRAC	1.579	0.042	1.418	1.665		
Time interval	2005, 2010, 2013, 2015, 2018, 2020					

Notes: P: Percentage.

**Table 4:** F-test and Hausman test of the model.

Model Selection	Dependent Variable	Statistical Test	Test Result	
Mixed or variable coefficient panel Models	LnC	F test	Chi-sq	10.76
			*p*-value	0
Fixed or random effects	LnC	Hausman test sigmamore	Chi-sq	74.52
			*p*-value	0

**Table 5:** Model results of GHG emissions and urban-rural settlements.

	Model_1		Model_2		Model_3	
Independent Variables	Coef.	Sig.	Coef.	Sig.	Coef.	Sig.
LnPOP	0.484	0.000	0.702	0.000	0.632	0.000
LnperGDP	0.524	0.000	0.480	0.000	0.474	0.000
LnIC	0.653	0.000	0.771	0.000	0.806	0.000
>P_UC			1.155	0.007	2.056	0.007
P_DUC						
P_SDUC						
P_SUA					1.282	0.013
P_LR			3.778	0.000	3.439	0.000
P_RA						
NP						
LPI						
LSI						
PARFRAC						
COHESION						
Constant	4.016	0.000	1.987	0.031	2.112	0.022
R-sq (within)	0.780		0.806		0.811	
F-statistic	295.620			213.680		263.300
Prob (F-statistic)	0.000		0.000		0.000	0.000
	Model_4		Model_5		Model_6	
Independent Variables	Coef.	Sig.	Coef.	Sig.	Coef.	Sig.
LnPOP	0.744	0.000	0.698	0.000	0.593	0.000
LnperGDP	0.467	0.000	0.473	0.000	0.476	0.000
LnIC	0.771	0.000	0.766	0.000	0.816	0.000
P_UC	1.330	0.001	1.236	0.008	2.223	0.005
P_DUC	8.660	0.089				
P_SDUC			−5.648	0.236		
P_SUA					1.688	0.028
P_LR	3.847	0.000	4.641	0.001	3.264	0.000
P_RA					5.458	0.453
NP						
LPI						
LSI						
PARFRAC						
COHESION						
Constant	1.489	0.071	1.955	0.038	2.187	0.014
R-sq (within)	0.812		0.809		0.812	
F-statistic		253.120		191.560		239.400
Prob (F-statistic)	0.000		0.000	0.000	0.000	0.000
N. of observation	126					
Confident interval	95%					

Note: The grey color indicates that the variable is insignificant in the model at 0.05 level. The following tables are the same.

**Table 6:** Model results of GHG emissions and landscape pattern indicators.

	Model_7		Model_8		Model_9	
Independent Variables	Coef.	Sig.	Coef.	Sig.	Coef.	Sig.
LnPOP	0.505	0.000	0.514	0.000	0.701	0.000
LnperGDP	0.522	0.000	0.527	0.000	0.481	0.000
LnIC	0.666	0.000	0.682	0.000	0.772	0.000
P_UC					1.156	0.006
P_DUC						
P_SDUC						
P_SUA						
P_LR					3.727	0.000
P_RA						
NP	0.001	0.071				
LPI						
LSI			0.029	0.003	0.002	0.855
PARFRAC						
COHESION						
Constant	3.743	0.000	3.418	0.000	1.973	0.031
R-sq (within)	0.7813	0.785		0.8062		
F-statistic		244.64		252.760	201.820	
Prob (F-statistic)		0		0.000	0.000	
N. of observation	126					
Confident interval	95%					

## Data Availability

Publicly available datasets were analyzed in this study. This data can be found here: [https://ghslsys.jrc.ec.europa.eu/ (accessed on 12 August 2022)].

## Appendix A

**Table A1:** Energy conversion coefficient of the studied year.

Energy	2005	2010	2013	2015	2018	2020
Raw coal (10,000 tons)	2.256	2.136	2.016	2.045	2.233	2.239
Washed coal (10,000 tons)	2.589	2.589	2.589	2.589	2.589	2.589
Other coal washing (10,000 tons)	1.511	1.511	1.511	1.524	1.553	1.553
Briquette (10,000 tons)	1.747	1.747	1.747	1.747	1.747	1.747
Coal gangue (10,000 tons)	0.000	0.575	0.633	0.633	0.633	0.575
Coke (10,000 tons)	2.946	3.067	3.067	3.067	3.067	3.067
Coke oven gas (100 million cubic meters)	8.954	8.328	8.328	8.328	8.328	8.328
Blast furnace gas (100 million cubic meters)	0.000	2.012	2.012	2.012	2.012	2.012
Converter gas (100 million cubic meters)	0.000	4.538	4.538	4.538	4.538	4.538
Other gas (100 million cubic meters)	5.117	3.178	3.178	3.178	3.178	3.178
Other coking products (10,000 tons)	3.644	3.644	3.644	3.644	3.644	3.644
Total oil products (10,000 tons)	3.107	3.082	3.100	3.118	3.117	3.100
Crude oil (10,000 tons)	3.067	3.067	3.067	3.067	3.067	3.067
Gasoline (10,000 tons)	3.158	3.158	3.158	3.158	3.158	3.158
Kerosene (10,000 tons)	3.158	3.158	3.158	3.158	3.158	3.158
Diesel (10,000 tons)	3.128	3.128	3.128	3.128	3.128	3.128
Fuel oil (10,000 tons)	3.067	3.067	3.067	3.067	3.067	3.067
Naphtha (10,000 tons)	0.000	3.220	3.220	3.220	3.220	3.220
Lubricating oil (10,000 tons)	0.000	3.036	3.036	3.036	3.036	3.036
Paraffin (10,000 tons)	0.000	2.930	2.930	2.930	2.930	2.930
Solvent oil (10,000 tons)	0.000	3.149	3.149	3.149	3.149	3.149
Petroleum asphalt (10,000 tons)	0.000	2.812	2.812	2.812	2.812	2.812
Petroleum coke (10,000 tons)	0.000	2.254	2.254	2.254	2.254	2.254
Liquefied Petroleum Gas (10,000 tons)	3.680	3.680	3.680	3.680	3.680	3.680
Refinery dry gas (10,000 tons)	3.373	3.373	3.373	3.373	3.373	3.373
Other petroleum products (10,000 tons)	2.814	2.855	2.855	2.855	2.855	2.855
Natural gas (100 million cubic meters)	21.840	21.840	21.347	21.347	21.038	20.955
Liquefied natural gas (10,000 tons) LNG	2.874	2.886	2.886	2.886	2.903	2.892
Heat (millions of kilojoules)	0.093	0.113	0.112	0.110	0.115	0.107
Electricity (100 million kWh)	7.912	7.140	6.606	5.752	6.061	5.354
Other energy sources (10,000 tons of standard coal)	/	0.000	0.000	0.000	0.000	0.000

Source: own elaboration.

**Table A2:** Guangdong Province Built-up Spatial Density Type Transition Matrix.

Area (km^2^)	2020 Type							
2005 Type	SRL	LR	RA	SUA	SDUC	DUC	UC	Total 2005
SRL	92,056	19,577	454	2101	261	6	250	114,705
LR	146	11,000	1342	7405	929	44	286	21,152
RA	-	189	1670	1173	589	15	2	3638
SUA	29	322	25	15,338	394	1085	1715	18,908
SDUC	-	64	80	884	1041	45	-	2114
DUC	-	-	10	506	120	2440	500	3576
UC	30	79	-	870	-	160	10,918	12,058
Total 2020	92,470	31,325	3583	28,296	3335	3795	13,685	177,667

Note: “-” means that this paired item was not detected according to the GHSL data.
